# Hazardous alcohol use in a sample of first episode psychosis patients in Singapore

**DOI:** 10.1186/s12888-019-2073-z

**Published:** 2019-03-15

**Authors:** Laxman Cetty, Shazana Shahwan, Pratika Satghare, Fiona Devi, Boon Yiang Chua, Swapna Verma, Helen Lee, Siow Ann Chong, Mythily Subramaniam

**Affiliations:** 1 Research Division, Institute of Mental Health, Buangkok Green Medical Park, 10 Buangkok View, Singapore, 539747 Singapore; 2 Department of Early Psychosis Intervention, Institute of Mental Health, Buangkok Green Medical Park, 10 Buangkok View, Singapore, 539747 Singapore; 3 Lee Kong Chian School of Medicine, Novena Campus, 11 Mandalay Road, Singapore, 308232 Singapore

**Keywords:** Hazardous alcohol use, Psychosis, Singapore, Hazardous drinking, AUDIT

## Abstract

**Background:**

Hazardous alcohol use has often been found to be more prevalent amongst psychiatric outpatients than the general population. Additionally, it has also been associated with poorer outcomes. The study aimed to investigate (1) the prevalence and (2) socio-demographic and clinical correlates of hazardous alcohol use, as well as (3) the relationship between hazardous alcohol use and quality of life in an outpatient sample with First Episode Psychosis (FEP) in Singapore.

**Methods:**

Baseline data (*N* = 280) was extracted from a longitudinal study investigating smoking and alcohol use amongst outpatients with FEP in a psychiatric hospital. Information on socio-demographics, hazardous alcohol use, and quality of life was collected through a self-report survey. Hazardous alcohol use was ascertained by total scores of 8 or higher on the Alcohol Use Disorders Identification Test (AUDIT). Data was analysed using logistic regression and linear regression analyses.

**Results:**

The prevalence of hazardous alcohol use over the past 12-month period was 12.9%. Those who had never smoked in their lifetime (vs current smokers) and those with a diagnosis of brief psychotic disorder (vs schizophrenia spectrum disorders) were found to have significantly lower odds of hazardous alcohol use. Hazardous alcohol use was also associated with lower negative symptom scores. Lastly, hazardous alcohol use was found to significantly predict lower scores on the physical health, social relationship and environment domains of quality of life.

**Conclusions:**

The association between hazardous alcohol use and lower negative symptom scores is a surprising finding that needs to be further explored. The significant impact of hazardous alcohol use in reductions in quality of life suggests that early screening and interventions could benefit patients with hazardous alcohol use and comorbid psychosis.

## Background

The Diagnostic and Statistical Manual (DSM) –IV [1] describes two distinct disorders of pathological alcohol use: alcohol abuse and alcohol dependence. The newer DSM-5 [2] however integrates the two disorders from DSM-IV into a single disorder: Alcohol Use Disorder (AUD) with mild, moderate, severe and other sub-classifications. The focus of the current paper; ‘hazardous drinking’, is defined as “a pattern of alcohol consumption that increases the risk of harmful consequences for the user or others” [3]. That is to say, it is a subthreshold disorder [4], not yet meeting the clinical threshold to be considered an AUD. In a meta-analytic review, Moyer and colleagues [5] suggest that brief interventions are effective in patients with less severe alcohol use, but not in those with more severe alcohol use. This seems to suggest that early interventions for people with hazardous alcohol use might be clinically advantageous.

People with schizophrenia have been found to be more likely to have alcohol problems than the general population [6–8]. Coupled with the knowledge that AUDs might further exacerbate the symptoms of schizophrenia, it seems important to further investigate alcohol use among people with schizophrenia or psychosis. First episode psychosis (FEP) in particular seems to mark a critical juncture in the treatment of schizophrenia. Past research seems to indicate that interventions soon after the onset of the first episode of psychosis are associated with better recovery (reduced symptoms and improved overall functioning) [9], especially when compared to traditional care [10]. Thus, this period also provides an opportunity for early intervention to treat comorbid physical and other mental health disorders including addictions.

AUDs have been found to be associated with a host of negative outcomes, particularly in patients with schizophrenia and psychosis. For example, individuals with FEP or schizophrenia, and comorbid AUD have been associated with greater positive symptoms [11–13]. Amongst associated socio-demographic factors, AUD has been found to be associated with being male in FEP [14] and lower education amongst individuals diagnosed with schizophrenia [15]. Additionally, being a current smoker was also associated with alcohol dependence among those with psychosis [16].

The prevalence of hazardous alcohol use seems to vary between Western and Asian countries. For example, the prevalence of hazardous alcohol use was reported to be 24% amongst a sample of psychiatric outpatients in Sweden [17] and 26% amongst a sample of outpatients with FEP in Canada [18]. On the other hand, the prevalence of hazardous alcohol use was reported to be 10.5% in a sample of psychiatric outpatients in Taiwan [19] and 5.5% in a sample of outpatients with schizophrenia in India [20]. As such, one might hypothesise the hazardous alcohol use in a population of FEP patients in Singapore to be lower than those reported in Western countries. While there have been previous studies conducted that have examined AUDs amongst the general population in Singapore [21], AUDs amongst inpatients of a general hospital in Singapore [22] and hazardous alcohol use amongst psychiatric outpatients [23] in Singapore, this is the first study to date to examine hazardous alcohol use among patients with FEP in Singapore.

This study aimed to investigate the prevalence of hazardous alcohol use amongst an outpatient population with FEP in Singapore. Additionally, we also sought to investigate socio-demographic and clinical correlates of hazardous alcohol use amongst those with FEP. Lastly, we aimed to investigate the relationships between quality of life and hazardous alcohol use.

## Methods

### Sample

The current paper reports data that was extracted from a longitudinal study, investigating smoking and alcohol use amongst outpatients who were seeking treatment at a tertiary level psychiatric hospital; the Institute of Mental Health (IMH), with the Early Psychosis Intervention Programme (EPIP), in Singapore. EPIP is a “comprehensive, integrated, and patient centred programme led by a multidisciplinary team of psychiatrists, psychologists, case managers, social workers, nurses and occupational therapists” [9]. Patients with FEP that is not substance-induced, with no prior or minimal treatment, no history of major medical or neurological illness, within the age range of 15–40 years are accepted into the programme. While the current study collected data from participants at three different time-points over the span of a year, only baseline data was examined in this paper (*N* = 280). For baseline data, participants who were within 3 months of admission into EPIP, able to understand English, and deemed clinically stable enough to participate in the study by a referring doctor or case manager, were approached by study team members, and recruited for the current study after obtaining informed consent. Study participants completed the self-report study questionnaires that were administered to them either on iPad or on paper. The questionnaires included measures reported below. The study obtained ethics approval from the National Healthcare Group (NHG) Domain Specific Review Board (DSRB) and the IMH Clinical Research Committee (CRC).

### Measures

#### Hazardous alcohol use

Hazardous alcohol use was measured using the 10-item brief screening instrument; the Alcohol Use Disorders Identification Test (AUDIT) [3]. The AUDIT was developed by the World Health Organization (WHO) as a screening instrument for hazardous and harmful alcohol use in primary health care. However the AUDIT has also been used on patients with FEP and has shown good reliability in detecting problem drinking [24]. The AUDIT covers the domains of alcohol consumption (items 1–3), drinking behaviours (items 4–6) and alcohol-related problems (items 7–10). A total score of 8 and above was used in the present paper to ascertain hazardous alcohol use (as suggested by Barbor and colleagues [3] for optimum sensitivity without compromising on specificity).

#### Quality of life

The World Health Organization Quality of Life – BREF (WHOQOL-BREF) is a 26 item questionnaire that was used to measure quality of life based on four domains: physical health (7 items), psychological health (6 items), social relationships (3 items), and environmental health (8 items) [25]. Two additional items scored separately, measured individuals’ ‘overall perception of quality of life’ and ‘overall perception of health’.

All 26 items on the WHOQOL-BREF are scored on a 5-point ordinal scale. A scaled score for each domain is obtained by multiplying the mean domain scores by 4 so that the scores may be directly comparable to those derived from the WHOQOL-100. Higher scale scores represent higher quality of life in the specific domain.

#### Socio-demographic information

Data on participants' socio-demographic background(age, gender, ethnicity, religion, highest education level attained, and current working status) was obtained from the self-administered questionnaire.

#### Clinical measures

Patient diagnosis was obtained by self-report and corroborated by patients’ medical records and clinician diagnosis. As EPIP regularly administers clinical assessments to patients at regular time intervals as part of its evaluation component [9], information from clinical assessments was obtained from the clinical database for the purpose of this study. Some of these clinical information included patients’ duration of untreated psychosis (DUP) (in months), positive, negative and general psychopathology scores from the Positive and Negative Syndrome Scale (PANSS) [26], as well as patients’ overall functioning scores from the Global Assessment of Functioning (GAF) instrument [1]. Details of its methods have been previously reported [9]. In brief, DUP was operationalised as the period of time (in months) between a patient’s onset of psychosis and formal diagnosis and treatment by the EPIP team. The PANSS was used to measure the severity of psychopathology. The GAF was used to assess overall levels of functioning (considering social, occupational and psychological functioning) of the patient.

#### Smoking history

Participants were asked if they were current smokers, social-smokers, ex-smokers or had never smoked. If they had ever smoked, they were then asked when was the last time they had smoked and the period of time they had been smoking.

### Statistical analysis

All statistical analyses were conducted using SPSS Statistics version 23. The prevalence of hazardous alcohol use and other study variables were reported using descriptive statistics. Mean and standard deviations were calculated for continuous variables, and frequencies and percentages for categorical variables. Multiple logistic regression analyses were conducted to examine socio-demographic and clinical correlates of hazardous alcohol use. Lastly, multiple linear regressions were conducted to establish associations between the quality of life domains and socio-demographic and clinical correlates. Statistically significant differences were evaluated at *p* < 0.05, using two-sided tests.

## Results

Data from 280 participants was analysed; 177 (63.2%) participants answered ‘yes’ to ever consuming alcohol beverages and completed the AUDIT questionnaire (Table 1). Total scores on the AUDIT ranged from 0 to 33. The past 12 month prevalence of hazardous alcohol use (total cut off score ≥ 8) amongst the study sample was found to be 12.9%.

**Table 1:** Sociodemographic and clinical characteristics of the sample

Variables	Hazardous Alcohol Use *(N = 36)*		No Hazardous Alcohol Use *(N = 244)*		Total *(N = 280)*	
	N	%	N	%	N	%
Age						
15–20	7	19.4	62	25.4	69	24.6
21–30	18	50.0	124	50.8	142	50.7
31–40	11	30.6	58	23.8	69	24.6
Gender						
Male	22	61.1	120	49.2	138	49.3
Female	14	38.9	124	50.8	142	50.7
Ethnicity						
Chinese	27	75.0	173	70.9	200	71.4
Malay	0	0.0	42	17.2	42	15.0
Indian	7	19.4	24	9.8	31	11.1
Others	2	5.6	5	2.0	7	2.5
Religion						
Christian	8	22.2	64	26.2	72	25.7
Buddhist	10	27.8	64	26.2	74	26.4
Muslim	2	5.6	54	22.1	56	20.0
Others	16	44.4	62	25.4	78	27.9
Education						
Secondary and below	13	36.1	64	26.2	77	27.5
Pre-tertiary/ Diploma/ Other	18	50.0	133	54.5	151	53.9
Tertiary	5	13.9	47	19.3	52	18.6
Work Status						
Working/ National Service	20	55.6	98	40.2	118	42.1
Student/ Housewife	5	13.9	73	29.9	78	27.9
Unemployed	11	30.6	67	27.5	78	27.9
Smoking Status						
Current Smoker	23	63.9	72	29.5	95	33.9
Never smoked	10	27.8	157	64.3	167	59.6
Social smoker or ex-smoker	3	8.3	14	5.7	17	6.1
Diagnosis						
Depression with psychotic features	2	5.6	11	4.5	13	4.6
Bipolar with psychotic features	2	5.6	8	3.3	10	3.6
Delusional Disorder	5	13.9	13	5.3	18	6.4
Brief psychotic disorder	1	2.8	31	12.7	32	11.4
Psychosis not otherwise specified	2	5.6	11	4.5	13	4.6
Schizophrenia Spectrum	21	68.3	128	52.5	149	53.2
Duration of untreated psychosis (in months) (*M* ± SD)	14.33	16.7	13.42	22.4	13.55	21.7
PANSS						
Positive (*M* ± SD)	22.8	5.6	21.8	6.1	21.9	6.0
Negative (*M* ± SD)	13.3	6.8	16.2	9.0	15.8	8.7
General psychopathology (*M* ± SD)	39.2	9.4	38.0	11.6	38.2	11.4
GAF (*M* ± SD)	43.3	10.2	44.5	12.4	44.3	12.1

Note. 6 missing responses for work status (2.1%). 1 missing response for smoking (0.4%). 45 missing responses for SCID diagnosis (16.1%). 44 missing responses for duration of untreated psychosis (DUP) (15.7%). 43 missing responses for PANSS scores (15.4%). 43 missing responses for GAF total score (15.4%)

### Socio-demographic and clinical correlates of hazardous alcohol use

No significant associations were found between the socio-demographic factors (age, gender, ethnicity, education, religion, and work status) and hazardous alcohol use (*p* > 0.05). Patients who never smoked in their lifetime (vs. current smokers) were found to have significantly lower odds of hazardous alcohol use (OR = 0.11, 95% CI [0.04, 0.34], *p* < 0.001) (Table 2).

**Table 2:** Sociodemographic and clinical correlates of hazardous alcohol use in persons with first-episode psychosis

Variables	OR	95% CI		*p*
		Lower	Upper	
Age				
15–20	Ref			
21–30	1.7	0.4	6.2	0.451
31–40	1.2	0.3	5.4	0.799
Gender				
Male	1.6	0.5	4.6	0.415
Female	Ref			
Ethnicity				
Chinese	Ref			
Malay	N.A	N.A	N.A	N.A
Indian	5.4	0.9	32.0	0.062
Others	17.6	0.5	611.2	0.113
Religion				
Christian	Ref			
Buddhist	1.8	0.5	6.5	0.392
Muslim	0.1	0.003	1.5	0.088
Others	1.0	0.3	3.8	0.982
Education				
Secondary and below	Ref			
Pre-tertiary/ Diploma/ Other	0.4	0.1	1.4	0.150
Tertiary	0.5	0.1	2.6	0.406
Work Status				
Working/ National Service	Ref			
Student/ Housewife	0.3	0.1	1.4	0.133
Unemployed	1.0	0.3	3.1	0.950
Smoking Status				
Current Smoker	Ref			
Never smoked	0.1	0.04	0.3	**0.000**
Social smoker or ex-smoker	1.3	0.2	8.4	0.769
Diagnosis				
Schizophrenia Spectrum	Ref			
Depression with psychotic features	5.4	0.5	60.4	0.170
Bipolar with psychotic features	1.2	0.2	10.3	0.845
Delusional Disorder	3.7	0.7	19.7	0.130
Brief psychotic disorder	0.1	0.004	0.7	**0.024**
Psychosis not otherwise specified	4.8	0.5	49.3	0.188
Duration of untreated psychosis (months)	1.0	1.0	1.0	0.770
PANSS				
Positive	1.1	1.0	1.2	0.067
Negative	0.9	0.9	1.0	**0.033**
General psychopathology	1.1	1.0	1.1	0.106
GAF	1.0	1.0	1.1	0.370

*OR* odds ratio, *95% CI* 95% confidence interval of odds ratio

Bold print highlights statistically significant odds ratio

Patients with a clinical diagnosis of brief psychotic disorder (vs. a diagnosis of schizophrenia spectrum disorders) were found to have significantly lower odds of hazardous alcohol use (OR = 0.05, 95% CI [0.00, 0.67], *p* = 0.024). Hazardous alcohol use was found to be associated with lower negative symptom scores (OR = 0.92, 95% CI [0.85, 0.99], *p* = 0.033) on the PANSS.

### Linear regression model with hazardous alcohol use predicting quality of life domains

Four separate linear regression models were run, investigating the relationship between hazardous alcohol use and the four domains of quality of life (physical, psychological, social relationships and environmental) as outcome variables, after adjusting for socio-demographic and clinical correlates (Table 3). For the model with the physical health domain of quality of life as the outcome variable, hazardous alcohol use was significantly associated with quality of life (β = − 1.79, *p* = 0.001). Similarly for the model with the social relationships domain of quality of life as the outcome variable, hazardous alcohol use was also significantly associated with quality of life (β = − 1.83, *p* = 0.003). Additionally, hazardous alcohol use was also significantly associated with the environmental domain of quality of life (β = − 1.17, *p* = 0.022). However, hazardous alcohol use was not significantly associated with the psychological health domain of quality of life (β = − 1.08, *p* = 0.067).

**Table 3:** Linear regression model of hazardous alcohol use predicting WHOQOL-bref

Variables	No Hazardous alcohol Use	Hazardous alcohol use					Model	
QOL Domain	M (± SD)	M (± SD)	B	SE	*t*	*p*	R^2^	Adjusted R^2^
Physical Health	70.4 (13.5)	62.5 (13.8)	−8.9	2.6	−3.5	**0.001**	0.249	0.153
Psychological Health	61.3 (15.8)	56.1 (13.6)	−5.4	2.9	−1.8	0.067	0.259	0.164
Social Relationships	65.3 (14.7)	55.0 (15.3)	−9.1	3.0	−3.1	**0.003**	0.250	0.154
Environment	66.8 (14.0)	59.4 (11.9)	−5.8	2.5	−2.3	**0.022**	0.292	0.202

No hazardous alcohol use is the reference group

Adjusted for age group, sex, ethnicity, religion, education, work status, diagnosis, smoking status, duration of untreated psychosis, positive, negative and general psychopathology scores of PANSS, and total GAF score

Bold print highlights statistically significant odds ratio

## Discussion

The 12-month prevalence of hazardous alcohol use in the population of patients with FEP in this study was found to be 12.9%. Having no history of smoking (vs with a history of smoking) and having a clinical diagnosis of brief psychotic disorder (vs schizophrenia spectrum) was found to reduce the likelihood of having hazardous alcohol use. Hazardous alcohol use was also associated with lower negative symptom scores on the PANSS scale. Lastly, hazardous alcohol use was found to significantly predict three out of the four domains of quality of life (physical health, social relationships and environment domains of quality of life).

The prevalence of hazardous alcohol use reported in this study (12.9%) is consistent with the trend from past literature that has indicated the prevalence of AUDs to be higher among a psychiatric population than in the general population [27]. While there are no studies that have reported to date hazardous alcohol use among the general population in Singapore, the prevalence of hazardous alcohol use reported in the current study can be compared against the prevalence of hazardous alcohol (measured by an AUDIT score of 8 or greater) amongst inpatients in a general hospital in Singapore (2.8%) [22]. One explanation for the higher prevalence of hazardous alcohol use amongst people with schizophrenia than in the general (non-psychiatric) population may be explained through the self-medication hypothesis. The self-medication hypothesis, postulates that people use substances such as alcohol as a means of self-regulating their distressing emotions [28, 29]. In a qualitative study involving a semi-structured interview design, participants with schizophrenia and a history of AUD were more likely to cite the use of alcohol to relieve symptoms of depression, and problems or worries than those without comorbid AUD [30]. Additionally, the reported prevalence in this paper is also consistent with the trend of lower prevalence of hazardous alcohol use in Asian countries compared to other Western countries as reported earlier in this paper. It is also consistent with generally lower prevalence of alcohol consumption in Asian countries compared to Western countries.

Having a clinical diagnosis of brief psychotic disorder was found to significantly reduce the odds of hazardous alcohol use. This is a new finding that has not been found before to the best of our knowledge. Having said that, a large portion of the study sample (*n* = 149, 53.2%) had a diagnosis of schizophrenia spectrum disorder. Comparatively, a much smaller portion of the sample had a diagnosis of brief psychotic disorder (*n* = 32, 11.4%). As such, any conclusions about the associations with a diagnosis of brief psychotic episode and hazardous alcohol use should be interpreted with caution. Future work in this area could consider performing similar studies with stratified sampling for diagnosis of the participants.

Hazardous alcohol use was found to be associated with lower negative symptom scores, indicative of fewer problems with social withdrawal, blunted affect, difficulties in abstract thinking and stereotyped thinking. While this might come across as a surprising finding, it is not new [31]. Tamo and colleagues found that patients with comorbid schizophrenia and substance use disorders (inclusive of AUDs) had lower negative symptom scores on the PANSS than those without a substance use disorder. In another study by Batki and colleagues [32], it was found that in a sample of participants with alcohol dependence, higher negative symptoms of schizophrenia were associated with lower frequency of alcohol drinking, alcohol cravings and quality of alcohol “high”. All the three study findings seem consistent and might be explained by the social nature of drinking behaviours especially in a younger sample group. Hence, individuals with less social withdrawal (lower negative symptom scores) might be better able to seek out opportunities to indulge in hazardous alcohol use. However, because of the cross-sectional nature of the current study, causal attributions cannot be made and it might well also be possible that hazardous alcohol use reduces negative symptom scores. Hence, further longitudinal research is still needed to validate this form of social explanation for drinking behaviours.

Additionally, individuals with a history of smoking were found to be more likely to report hazardous alcohol use. This finding is consistent with previous research indicating the frequent co-occurrence of smoking and excess alcohol use [23, 33, 34]. One study suggests that people who consider themselves to be risk takers were more likely to share a genetic risk with schizophrenia and be smokers [35]. This might indicate that risk taking behaviours such as hazardous alcohol use and smoking might have a genetic link with schizophrenia. In terms of health outcomes, consuming excessive alcohol and smoking seems to be particularly detrimental. A longitudinal study by Hart and colleagues [36] found that men who both smoked and drank more than 15 units of alcohol per week were at the highest risk of all causes of death investigated in the study. This highlights the need to screen for the co-occurrence of excessive alcohol use and cigarette smoking among individuals with FEP and deliver appropriate interventional services as this group seems particularly vulnerable to worse health outcomes.

Importantly, hazardous alcohol use was able to predict three out of the four domains of quality of life (physical health, social relationships and environmental domains). This is a significant finding that highlights the seriousness of hazardous alcohol use in the current sample. The physical health domain of the WHOQOL-BREF includes items on pain, energy levels, mobility, sleep and capacity for daily living activities. The association between alcohol consumption and poorer physical health has been generally well-established in research [37], including disturbances to sleep [38] and impaired daily activities [39]. The social relationships domain of the scale includes items on satisfaction with personal relationships and the social support being received. Additionally, the environmental domain of the WHOQOL-BREF includes items on the home environment, access to transport, opportunity for leisure activities, satisfaction with finances and access to health services. Poor social and physical environments have often been associated with alcohol use amongst those with schizophrenia and psychosis with many living in poverty, and limited opportunities, as well as facing issues of stigma and segregation due to the illness, often exacerbated with the AUDs [40]. This is in addition to the already established findings that AUDs adversely affect marital satisfaction and stability, as well as the family institution [41]. The findings from the current paper suggest the pervasive negative effect of hazardous alcohol use in people with psychosis and highlight the need for added support, focused on individuals with psychosis living in worse physical environments, with problematic relationships and with comorbid hazardous alcohol use.

The mean values reported in the current study of the quality of life domains can be directly compared to an earlier study by Cheung and colleagues in Singapore [42]. Their study reported mean quality of life scores segregated by the four domains in a sample comprising those from the general population (*N* = 892) as well as from tertiary hospitals with varying health conditions (diabetes, heart disease, mental illness etc.) (*N* = 424). The overall mean scores for quality of life from the sample in the current study was lower for all the four domains of quality of life (physical health, psychological health, social relationships and environment domains), when compared to the pooled sample in the study by Cheung et al. Given that the sample population in the current study have psychosis and are within the first 3 months of diagnosis, it is an expected finding. However, it is important to note that the hazardous alcohol use group in particular in the current study have much lower scores of quality of life which is a cause for concern and an opportunity for early intervention.

The prevalence of hazardous alcohol use amongst patients with FEP reported in the current study can be compared to a similar study which examined hazardous alcohol use amongst psychiatric outpatients in Singapore (albeit with a sample population with a diagnosis of schizophrenia or depression) [23]. The prevalence of hazardous alcohol use in the current study among outpatients with FEP was found to be almost double (12.9%) compared to patients with schizophrenia (6.4%). This finding again supports the self-medication hypothesis, especially given that patients with FEP in our study were recruited within 3 months of diagnosis. It might be hypothesised that patients with a more recent onset of psychosis might initially use alcohol to cope with the distressing symptoms of psychosis but might grow less reliant on it, with psychiatric treatment filling in that function. However, the consistent finding of hazardous alcohol being associated with poorer quality of life in the physical health domain in both studies suggest that the effects of hazardous alcohol use on physical health does not get better even with continued psychiatric treatment.

The findings of this paper highlight the need for screening, intervention and appropriate referral for patients. Literature has indicated positive results thus far on the value of early screening and interventions for problem drinking and hazardous alcohol use [43]. For example, in a study by Archie and colleagues [18], hazardous alcohol use was found to be significantly lower after 12 months than at baseline after early intervention amongst a sample of first-episode psychosis patients. Another study [44] indicated that when hazardous alcohol was reduced (after a targeted intervention amongst a sample of psychiatric patients), anxiety and depressive symptoms improved significantly faster than patients without the intervention.

### Strengths and limitations

The current paper’s strengths lie in that it is the first paper to our knowledge that has examined the associated factors and clinical outcomes of hazardous alcohol use among a study sample with FEP, as well the relationship with the quality of life domains. While a lot of previous research has often subsumed alcohol use and abuse under “substance use disorders”, this paper builds on current limited research on the prevalence of specifically hazardous alcohol use amongst the FEP population. Furthermore, the paper included a multi-ethnic population of patients with FEP, building on research on existing research in hazardous alcohol use amongst the FEP population in Asia.

The findings reported in this paper should be interpreted in accordance with the study’s limitations. Hazardous alcohol use in this paper was measured with a self-report instrument (the AUDIT scale). This permits social desirability bias with participants possibly under-reporting their alcohol consumption. This would mean that the true prevalence of hazardous alcohol use in the study sample could be higher than reported. However, this was minimised by using a self-report method of data collection (as opposed to needing to interact with an interviewer). Additionally, we used a cut-off of 8 points on the AUDIT scale, for both men and women, for identification of hazardous alcohol use. This cut-off point was deliberately chosen to make a direct comparison with Tay and colleague’s study [22] which also assessed hazardous alcohol amongst inpatients in a general hospital in the same country (Singapore). However, a lower cut-off point (6 points or more) has been suggested for women in some studies to be more appropriate [45, 46]. This would mean that the true prevalence of hazardous alcohol use in the study would likely be higher. Participants might also have difficulty remembering the number of units of alcohol they had consumed due to memory impairments in forming new long term memories, particularly after consuming large amounts of alcohol and if consumed rapidly [47]. This might also have influenced the prevalence of hazardous alcohol use in the study sample. The number of hazardous drinkers (*n* = 36) found in the sample is small and the results from the multiple logistic and linear regression analyses must be interpreted with caution. Lastly, due to the cross-sectional nature of the study design, temporal relationships cannot be drawn about comorbid hazardous alcohol use and the onset FEP.

## Conclusions

The current paper details the prevalence and correlates of hazardous alcohol use in an outpatient sample with FEP in Singapore. Additionally, it also highlights the predictive value of hazardous alcohol use on one’s quality of life. The findings suggest a need for routine screenings for hazardous alcohol use amongst FEP patients. With increasing evidence that brief interventions targeted at these patients could alleviate some of the negative outcomes, mental health service providers should strongly consider its implementation in the clinical settings.

## Abbreviations

AUD: Alcohol Use Disorder
AUDIT: Alcohol Use Disorders Identification Test
CRC: Clinical Research Committee
DSM: Diagnostic and Statistical Manual
DSRB: Domain Specific Review Board
DUP: Duration of Untreated Psychosis
EPIP: Early Psychosis Intervention Programme
FEP: First Episode Psychosis
GAF: Global Assessment of Functioning
IMH: Institute of Mental Health
NHG: National Healthcare Group
PANSS: Positive and Negative Syndrome Scale
WHO: World Health Organization
WHOQOL-BREF: World Health Organization Quality of Life – BREF
